# Dynamic interactions among anxiety, emotional stability, and mindfulness: a cross-lagged panel network analysis

**DOI:** 10.3389/fpsyg.2026.1740018

**Published:** 2026-07-21

**Authors:** Yuntai Wang, Jiangtao Han, Yukun Mei, Shiyi Lin, Guodong Gong, Fengqiong Zheng, Songkai He, Jian Sun

**Affiliations:** 1 Xihua University, Chengdu, China; 2 Guangdong Key Laboratory of Mental Health and Cognitive Science, Center for Studies of Psychological Application, School of Psychology, South China Normal University, Guangzhou, China; 3 Xiamen University, Xiamen, China; 4 Yunnan Normal University, Kunming, China; 5 Sichuan University of Science and Engineering, Zigong, China

**Keywords:** anxiety, cross-lagged panel network design, emotional stability, mindfulness, predictive associations

## Abstract

**Background:**

Anxiety is one of the most prevalent global mental health disorders, with its incidence having risen substantially since the COVID-19 pandemic and remaining elevated in the post-pandemic era. According to the process model of emotion regulation, mindfulness may serve as an early-stage cognitive change strategy that enhances emotional stability and ultimately reduces anxiety. While previous research has demonstrated robust interconnections among anxiety, emotional stability, and mindfulness, the majority of studies have relied on cross-sectional designs and aggregate scores, neglecting their longitudinal, item-level dynamic relationships.

**Method:**

This study collected data from 547 college students (*M*_age_ ± *SD* = 20.11 ± 1.20) across three time points and utilized a cross-lagged panel network (CLPN) approach to analyze item-level temporal dynamics. The goal was to build network models of anxiety, emotional stability, and mindfulness, and to investigate how these constructs were temporally interrelated at the item level in an exploratory manner.

**Result:**

Mindfulness items had higher out-Expected influence (OEI), whereas anxiety symptoms had stronger in-Expected Influence (IEI), with anxiety and emotional stability having the strongest bridge effects. Specifically, mindfulness components “Thought Acceptance” (M7) and “Nonjudgmental Awareness” (M8) exerted significant effect on anxiety and emotional stability. Anxiety symptoms were highly responsive to other symptoms, which undermined emotional stability. The network analysis revealed complex temporal and predictive associations among anxiety, emotional stability, and mindfulness.

**Conclusion:**

This study is the first investigation to reveal the longitudinal dynamic connections between anxiety, emotional stability, and mindfulness at the item level, consequently provided longitudinal support consistent with the emotion regulation process model.

## 1 Introduction

Anxiety is one of the most prevalent mental health disorders worldwide, with the global prevalence increasing by 23.2–28.0% after the COVID-19 pandemic (Santomauro et al., 2021), and accumulating evidence suggests that elevated anxiety levels have persisted into the post-pandemic period (Aknin et al., 2022; Son et al., 2020). Anxiety exerts an enormous burden on individuals and society, closely associated with multiple diseases and adverse health outcomes (Karami et al., 2023; Shahbazi et al., 2022; Storer et al., 2023). However, individuals exhibit heterogeneous emotional response patterns when confronted with anxiety triggers (Duschek et al., 2020; Rampino et al., 2019). Emotional stability, a key component of the Big Five personality traits, denotes the intensity and variability of an individual’s emotional reactions in response to stress, obstacles, and negative emotions (Osimo et al., 2021). High emotional stability is associated with stronger emotional resilience and is vital in mitigating the intensification of anxiety (Grisanzio et al., 2018; Lee et al., 2024). Understanding how emotional stability is developed and maintained requires a theoretical framework of emotion regulation. According to the process model of emotion regulation (Gross, 2002), the most effective regulation occurs during the early “cognitive change” stage, when individuals can alter their appraisal of emotion-eliciting situations before a full emotional response unfolds. This framework suggests that examining the regulatory processes that precede emotional stability—and the anxiety outcomes that follow its disruption—may yield critical insights for intervention.

Within this framework, mindfulness has been increasingly recognized as a key cognitive change strategy. Mindfulness, defined as a conscious and nonjudgmental awareness of the present moment, facilitates profound cognitive reappraisal by enabling individuals to detach from automatic ideas and embrace a more expansive, accepting viewpoint on interior experiences, rather than repressing emotions (Creswell, 2017; Epstein, 1999). In accordance with James J. Gross’s Process Model of Emotion Regulation, we assert that an individual’s state of mindfulness, by fostering acceptance-based cognitive transformation, can enhance emotional stability and ultimately reduce anxiety.

In fact, numerous empirical investigations have evidenced bidirectional relationships among anxiety, emotional stability, and mindfulness, as well as the emotional regulation function of mindfulness at the individual level. Studies demonstrate that mindfulness significantly improves emotional stability and effectively reduces anxiety symptoms (Al-Refae et al., 2021; Treves et al., 2022). Investigation on heart rate variability (HRV) indicates that enhanced mindfulness boosts emotional stability by regulating autonomic nervous system function (S. Lee et al., 2025). Simultaneously, mindfulness acceptance training (MAT) significantly diminishes individual anxiety symptoms and improves emotional self-regulation effectiveness (Yang et al., 2022). Regarding emotional stability and anxiety, research shows that individuals with lower emotional stability exhibit more significant anxiety symptoms (Akram et al., 2019). Neurobiological evidence indicates that emotion regulation and cognitive functions engage overlapping brain networks, with the prefrontal cortex being pivotal for emotional stability and anxiety (Horato et al., 2022). Current research, however, lacks longitudinal designs and mostly analyzes the interrelationships among these variables using aggregate scores, neglecting the complex associations at the item level.

Although mindfulness and emotional stability are both relevant to emotion regulation, they are theoretically and operationally distinguishable. Mindfulness is primarily conceptualized as a metacognitive attentional process involving present-centered awareness, nonjudgmental observation, and acceptance of internal experiences (Baer et al., 2006; Reina and Kudesia, 2020). In contrast, emotional stability refers to a relatively enduring dispositional tendency to maintain affective equilibrium and resist excessive emotional fluctuation in the face of stress (DeNeve and Cooper, 1998; Schimmack, 2003). While mindfulness may function as a regulatory mechanism that facilitates emotional stability, the two constructs differ in their psychological level of analysis: mindfulness represents a process-oriented, awareness-based capacity, whereas emotional stability reflects a trait-level outcome of effective affect regulation. Examining their temporal interplay at the item level may therefore help clarify how awareness-related regulatory processes and stable affective functioning are dynamically linked over time.

In recent years, network analysis has been increasingly adopted in the mental health field, conceptualizing psychological conditions as networks of interconnected symptoms. Unlike traditional approaches that conceptualize mental health conditions as unified entities with distinct etiological pathways (Borsboom, 2017), this framework posits that conditions are driven by the mutual activation and dynamic evolution of symptoms (Borsboom and Cramer, 2013). Within the network analysis, mental disorders are depicted as networks. These networks comprise nodes that denote symptoms or indicators and edges that indicate the interactions among them. Typically, nodes associated with different conditions are grouped into separate communities (Epskamp et al., 2012). The conception of psychopathology as a network enables the identification of central symptoms and enhances the understanding of their dynamic connections.

Traditional cross-sectional studies cannot reveal potential directional temporal relationships between symptoms, whereas the Cross-Lagged Panel Network (CLPN) is a recently reported novel method that can identify autoregressive effects, cross-lagged effects, and bridge centrality all at once (Epskamp et al., 2012). By analyzing autoregressive and cross-lagged effects, the CLPN model can elucidate predictive associations between symptoms, identify symptoms with high out-Expected Influence (OEI; strong predictive effects on other symptoms) and those with high in-Expected Influence (IEI; high susceptibility to prediction by other symptoms). This provides precise targets for intervention, thereby disrupting the vicious cycle of comorbid networks (Epskamp et al., 2012; Fried and Cramer, 2017). The CLPN approach is well-suited for longitudinal panel data collected across two or more waves, allowing for the integrated network modeling of anxiety, emotional stability, and mindfulness symptoms.

Current studies support bidirectional relationships among anxiety, emotional stability, and mindfulness. Nonetheless, prior research is limited by two primary constraints: first, a dependence on cross-sectional data, which hinders the elucidation of dynamic relationships at the item level; and second, an absence of systematic modeling of the multivariate network dynamics. To address these constraints, the present study innovatively employs network analysis, specifically utilizing the Cross-Lagged Panel Network (CLPN) technique to construct two CLPNs (i.e., T1- > T2 and T2- > T3) that model the dynamics of anxiety, emotional stability, and mindfulness. This methodology aims to pinpoint key indicators with significant intervention potential and to investigate the temporal and directional associations at the item level among these three mental conditions.

## 2 Method

### 2.1 Participants

This study used a three-wave convenience sampling design at a public university in Sichuan Province, China. Data were collected in May 2025 (T1), June 2025 (T2), and July 2025 (T3) through the Wenjuanxing platform^1^ (Etikan et al., 2015). Participants reported their age, gender (1 = male, 2 = female), ethnicity (1 = Han, 2 = ethnic minority), and marital status (1 = married, 2 = single).

To ensure data quality and accuracy, stringent quality control measures were implemented during the questionnaire administration and data processing phases. Because the survey was administered online via the Wenjuanxing platform, data entry and compilation were automated, effectively eliminating manual data entry errors. Furthermore, during the questionnaire design phase, attention checks (i.e., logical trap questions) were embedded to identify perfunctory, careless, or random responding. These checks consisted of simple, straightforward questions with clear, unambiguous, and unique correct answers. If a participant selected an illogical or obviously incorrect answer to these checks, their questionnaire was deemed invalid and excluded from the dataset to maximize data authenticity and reliability.

Following these quality control procedures, across the three waves, 645 participants provided valid responses at T1, 576 completed T2, and 550 completed T3. Attrition between waves was mainly due to some students being close to graduation and therefore being unable to participate in subsequent assessments because of competing demands such as thesis completion, internship arrangements, job preparation, and graduation procedures. After matching responses across waves using participants’ phone numbers, 547 participants had complete data on anxiety, emotional stability, and mindfulness at all three time points and were retained for the final analyses, corresponding to an 84.80% retention rate from T1 to T3. The discrepancy between the T3 sample (*n* = 550) and the final analytic sample (*n* = 547) was due to missing values on key study variables. Therefore, the final analyses were conducted on the complete-case sample using listwise deletion.

Before participation, written informed consent was obtained from all participants and, where applicable, their guardians. The consent form described the study purpose, emphasized the voluntary nature of participation and the right to withdraw without penalty, and assured data anonymity in accordance with the Declaration of Helsinki. Formal permission for the study was obtained from the local education authority and the university administration. In addition, all study materials and procedures were approved by the Scientific Review Group of Xihua University (Institutional Review Board No. XH25071501).

### 2.2 Measures

The selection of brief instruments in this study was primarily guided by the practical need to minimize participant response burden in a three-wave longitudinal design. Repeated administration of lengthy questionnaires across multiple time points can lead to participant fatigue, reduced response quality, and increased attrition. Brief, well-validated instruments were therefore prioritized to balance measurement adequacy with data quality and participant retention.

#### 2.2.1 Emotional stability

The Chinese Big Five Personality Inventory Brief Version (CBF-PI-B) is a prevalent tool in personality psychology, exhibiting strong reliability and validity within Chinese populations, encompassing five fundamental dimensions: Emotional Stability, Extraversion, Agreeableness, Conscientiousness, and Openness (Zhang et al., 2019). For the present study, we utilized the 3-item Emotional Stability subscale from this inventory to minimize participant response burden in a three-wave longitudinal design. Each item was rated on a 6-point Likert scale, ranging from 1 (strongly disagree) to 6 (strongly agree). The Cronbach’s *α* coefficients for this subscale were excellent at T1 (*α* = 0.95), T2 (*α* = 0.95) and T3 (*α* = 0.97), indicating high internal consistency across all three measurement waves. The construct validity of the Emotional Stability subscale has been established in prior research (Zhang et al., 2019).

#### 2.2.2 Mindfulness

Participants’ mindfulness levels were assessed using the 9-item Multidimensional State Mindfulness Questionnaire (Blanke and Brose, 2016). The Chinese adaptation of this scale has shown sound psychometric properties among Chinese college students (Zhou et al., 2021). The Cronbach’s *α* coefficients for this scale were excellent across T1 (*α* = 0 0.97), T2 (*α* = 0.98), and T3 (*α* = 0.96), indicating high internal consistency across measurement waves.

#### 2.2.3 Anxiety

The Chinese Version Generalized Anxiety Disorder 2-item (GAD-2) scale was employed to measure anxiety. This scale has demonstrated good reliability in Chinese populations (Luo et al., 2019). Responses range from 0 (not at all) to 3 (nearly every day). In this study, the GAD-2 exhibited excellent internal consistency, as indicated by Cronbach’s *α* coefficients T1 (*α* = 0 0.90), T2 (*α* = 0.90), and T3 (*α* = 0.92).

### 2.3 Statistical analysis

#### 2.3.1 Network estimation and visualization

All statistical analyses for this study were conducted using *R* software (Version 4.4.2). All symptom scores were standardized into *z*-scores to ensure uniform scaling for subsequent analyses. Network nodes were defined as individual items from the three measurement instruments: two items from the GAD-2, three items from the Emotional Stability Scale, and nine items from the Mindfulness Scale, yielding a total of 13 nodes per wave. The *glmnet* package was employed to construct the CLPN via regression analysis (Wysocki et al., 2017). The model incorporated LASSO (Least Absolute Shrinkage and Selection Operator) sparse regularization to identify significant cross-lagged effects and prune redundant connections (Friedman et al., 2008). A 10-fold cross-validation procedure was used to optimize the LASSO regularization tuning parameter (*λ*), balancing model complexity and generalizability (Friedman et al., 2010). Network visualization was accomplished with the *qgraph* package (Epskamp et al., 2012). Nodes correspond to symptoms, and directed edges represent cross-lagged predictive pathways. The visual convention uses blue edges for positive regression weights and red edges for negative weights.

The *bootnet* package was employed to compute centrality indices, specifically in-Expected Influence (IEI) and out-Expected Influence (OEI), to elucidate the directional pathways within the network. Symptoms with high OEI are those that, when present at an earlier wave, are strong predictors of all other symptoms at the next wave. A high IEI value denotes a symptom that is highly predictable from the aggregate of all other symptoms measured at the previous time point. Edge weights (regression coefficients) greater than 0 represent positive associations, while values less than 0 indicate negative associations; a value of 0 denotes the absence of a relationship (Jones et al., 2021). Result visualizations were created using the *ggplot 2* package (Villanueva and Chen, 2019). Key network indices, including centrality metrics (e.g., edge weights, expected influence), served to assess inter-nodal connection strength and the relative impact of individual nodes in the network.

#### 2.3.2 Accuracy and stability estimation

The *bootnet* package was employed to evaluate the stability and accuracy of the network models (Epskamp et al., 2018). First, a non-parametric bootstrap with 1,000 bootstrap samples was applied to the original data to estimate the 95% confidence intervals (*CI*s) for the edge weights, thereby assessing the network’s stability. Wide bootstrap *CI*s indicate low precision and cast doubt on the stability of the respective edges. Next, the accuracy of centrality metrics was evaluated via a case-drop bootstrap, which iteratively removes participants to calculate the Correlation Stability Coefficient (*CS*). This coefficient indicates the maximum proportion of the sample that can be excluded such that the correlation between centrality indices from the original network and those from the perturbed networks remains above 0.70. Following the standardized recommendations of Epskamp et al. (2018), a CS coefficient > 0.25 indicates acceptable stability, and a value > 0.50 suggests high robustness.

Additionally, we tested for significant differences in centrality indices and edge weights, with a specific focus on the interconnections among anxiety, emotional stability, and mindfulness, using a non-parametric bootstrap procedure for edge weight difference testing. Difference testing was conducted using the minimum confidence interval approach, with the alpha level corrected via the *Bonferroni* method to mitigate the inflation of *Type I* error due to multiple testing. Moreover, the differences in network edge weights were visualized. By plotting a difference network and a heat map, the disparities and directional patterns in centrality metrics and edge weights were illustrated. In the difference network plot, the color intensity of the edges reflects the significance of the weight differences, whereas the heat map uses a color gradient to represent the contrast in edge weights.

## 3 Results

### 3.1 Demographic characteristics

The participants were primarily female, aged 17 to 34 years (*Mean* ± *SD* = 20.11 ± 1.20). The majority were of Han ethnicity, possessed a senior high school diploma or a bachelor’s degree, and were single. Additional demographic details are elaborated in Table 1.

**Table 1:** Demographic Information (*N* = 547).

	Frequency	Percentage
Gender		
Male	218	39.85%
Female	329	60.15%
Ethnic		
Han	483	88.30%
Minor	64	11.70%
Education background		
High school or below	12	2.19%
Bachelor’s degree	264	48.26%
Master’s degree	268	48.99%
Doctoral degree	3	0.55%
Spouse		
Married	67	12.25%
Single	480	87.75%

### 3.2 Network inference

**Figure 1:**
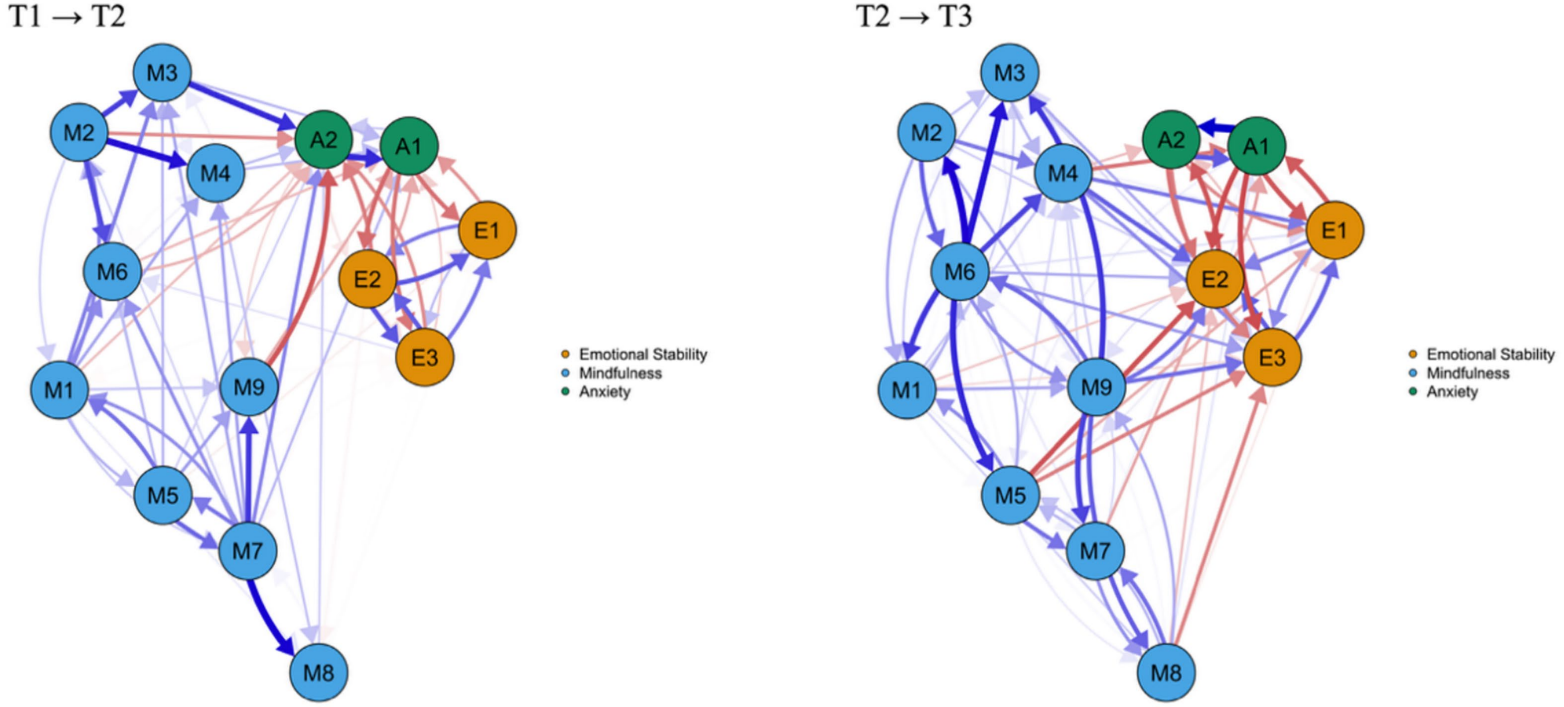
Cross-lagged panel network of anxiety, emotional stability, and mindfulness. Blue edges represent positive cross-lagged associations, and red edges represent negative cross-lagged associations. The thickness of the edges represents the relative strength of the associations, with thicker edges indicating stronger relationships. For visual clarity, the autoregressive edges are hidden. E1 = Not concerned with trivialities, E2 = Inner peace, E3 = Optimism, M1 = Conscious task engagement, M2 = Full task focus, M3 = Present-moment focus, M4 = Present-moment attention, M5 = Openness to experience, M6 = Full activity absorption, M7 = Thought acceptance, M8 = Nonjudgmental awareness, M9 = Acceptance of performance, A1 = Nervousness, A2 = Uncontrollable worrying.

**Figure 2:**
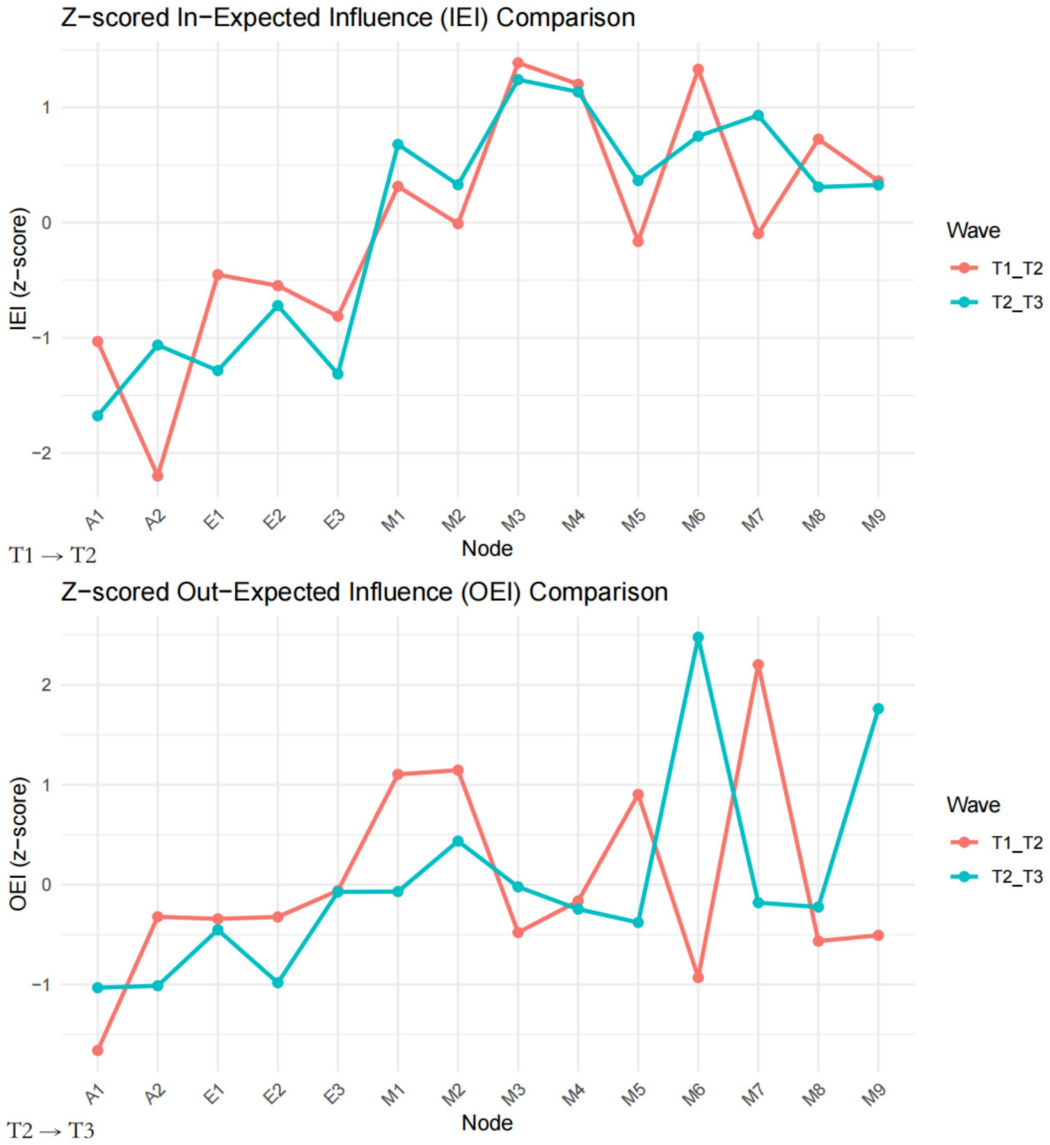
Centrality estimates of Anxiety, Emotional stability, and Mindfulness (*z* values). High values indicate more centrality. E1 = Not concerned with trivialities, E2 = Inner peace, E3 = Optimism, M1 = Conscious task engagement, M2 = Full task focus, M3 = Present-moment focus, M4 = Present-moment attention, M5 = Openness to experience, M6 = Full activity absorption, M7 = Thought acceptance, M8 = Nonjudgmental awareness, M9 = Acceptance of performance, A1 = Nervousness, A2 = Uncontrollable worrying.

Figure 1 depicts the CLPNs estimated for T1 → T2 and T2 → T3. To improve visual clarity, autoregressive pathways are omitted from this picture; complete networks incorporating all autoregressive effects are available in Supplementary Figure 1. Figure 2 displays the network centrality metrics, comprising OEI and IEI, for the T1 → T2 and T2 → T3 networks. The quantitative values of these centrality metrics are provided in Supplementary Table 1.

First, as illustrated in Supplementary Table 2, 3, the most significant connections between the emotional stability, mindfulness, and anxiety domains were M7 (Thought acceptance) → M8 (Nonjudgmental awareness) in the T1 → T2 network and A1 (Nervousness) → A2 (Uncontrollable worrying) in the T2 → T3 network, respectively. The most robust connections between the emotional stability, mindfulness, and anxiety communities were A1 (Nervousness) → E2 (Inner peace) in the T1 → T2 network and A1 (Nervousness) → E3 (Optimism) in the T2 → T3 network, respectively.

Secondly, as illustrated in Figure 2, the node exhibiting the highest absolute OEI value in the T1 → T2 network was M7 (Thought acceptance; OEI = 2.18), whereas in the T2 → T3 network, it was M6 (Full activity absorption; OEI = 2.45), signifying that these symptoms were the most potent in activating other nodes within their respective networks. Furthermore, the node exhibiting the highest absolute IEI value in the T1 → T2 network was A2 (Uncontrollable worrying; IEI = −2.20), for the T2 → T3 network, it was A1 (Nervousness; IEI = −1.68), indicating that these symptoms were the most responsive to the effects of other nodes within the network.

### 3.3 Network stability and accuracy

The 95% bootstrapped *CI*s for the edge weights in both the T1 → T2 and T2 → T3 CLPNs were narrow to moderate (Supplementary Figure 2), and the strongest and weakest edges differed significantly (see Supplementary Figure 3), indicating accurate estimation of these edges (Epskamp et al., 2018). Results from the case-dropping bootstrap analysis (Supplementary Figure 4) indicated acceptable stability for most indices: CS coefficients for IEI were 0.52 (T1 → T2) and 0.67 (T2 → T3), demonstrating good robustness. Values for OEI (CS = 0.28 for both networks) and edge weights (CS = 0.28 and 0.36) fell within the acceptable range (Kim and Lee, 2022). The results of the centrality difference tests are presented in Supplementary Figure 5, 6.

## 4 Discussion

Using a Cross-Lagged Panel Network (CLPN) design, this study provides initial evidence for the item-level temporal dynamics among anxiety, emotional stability, and mindfulness from a longitudinal perspective. In summary, the results indicated that symptoms associated with mindfulness exhibited a greater out-Expected Influence (OEI), but anxiety-related symptoms showed a higher in-Expected Influence (IEI). This indicates that mindfulness may function as a psychological regulatory mechanism and serves as a crucial intervention target for modifying future psychological states, while anxiety is predominantly governed by emotional regulation processes. These findings collectively offer compelling evidence illuminating the dynamic mechanisms that connect mindfulness, emotional stability, and anxiety symptoms.

Firstly, ‘Thought acceptance’ (M7) and ‘Full activity absorption’ (M6) emerged as nodes with high out-Expected Influence (OEI) across the three times intervals, suggesting that these facets of mindfulness may be key elements in maintaining emotional stability and alleviating anxiety (Han et al., 2023). Thought acceptance emphasizes a nonjudgmental stance toward one’s own thoughts and feelings, whereas full absorption requires individuals to focus their attention on present-moment experiences, thereby avoiding excessive worry about the past or future (Al-Refae et al., 2021; Han et al., 2023). Consistent with prior research, accepting one’s thoughts helps individuals reduce self-criticism and over-identification with negative emotions, thereby promoting emotional stability (Patel et al., 2018). As another core component of mindfulness, the mechanism of full absorption primarily lies in attentional control and emotion regulation. Research indicates that full absorption aids individuals in better identifying and managing emotions, reducing the automaticity and intensity of emotional responses, which is consistent with our findings (Borgdorf et al., 2024; Pires et al., 2018). Within Gross’s process model of emotion regulation, acceptance facilitates cognitive change that disrupts automatic negative emotional responses, ultimately bolstering emotional resilience (Gross, 2002). This study’s findings provide longitudinal evidence consistent with the theoretical proposition, suggesting that enhancing mindfulness traits may be temporally associated with indirect alleviation of anxiety through the pathway of improved emotional stability. Moreover, the observed temporal associations between mindfulness items (e.g., Thought acceptance (M7) and Full activity absorption (M6)) and emotional stability indicators further support their conceptual distinction: mindfulness items functioned primarily as temporal antecedents (high out-Expected Influence), whereas emotional stability items functioned as temporal consequences (high in-Expected Influence), suggesting that mindfulness and emotional stability play different dynamic roles within the network rather than being redundant reflections of a single latent process.

These findings carry important implications for clinical intervention design. The identification of ‘Thought acceptance’ (M7) and ‘Full activity absorption’ (M6) as the most influential temporal antecedents in the network suggests that these specific facets of mindfulness represent high-priority targets for preventive and therapeutic programs. Clinically, interventions that specifically cultivate nonjudgmental acceptance of thoughts—such as Acceptance and Commitment Therapy (ACT; Hayes et al., 2006) and Mindfulness-Based Cognitive Therapy (MBCT; Riemann et al., 2016)—may be particularly effective in disrupting the temporal pathway from low mindfulness to emotional instability and subsequent anxiety escalation. Unlike broad-spectrum mindfulness interventions that target mindfulness as a unitary construct, the present item-level findings suggest that intervention efficiency may be enhanced by prioritizing these two specific components, particularly in populations at risk for anxiety-related emotional dysregulation.

Secondly, the anxiety symptoms “Nervousness” (A1) and “Uncontrollable worrying” (A2) exhibited high absolute values of IEI across both networks. However, the IEI values for these nodes were negative, indicating that these anxiety symptoms are susceptible to being diminished by the influence of mindfulness and emotional stability. This finding provides novel empirical support and practical direction for interventions targeting anxiety symptoms. Aligning with existing literature, “Nervousness” and “Uncontrollable worrying” — central symptoms of anxiety — exhibit intricate interplay within the anxiety symptom network (Cai et al., 2023). Regarding the underlying psychological mechanism, uncontrollable worrying is conceptualized as a coping strategy aimed at reducing uncertainty by proactively addressing potential threats (Gústavsson et al., 2021).

In addition, in both created CLPNs, the most significant bridge edges originated from “Nervousness” (A1) to indicators of emotional stability (E2 (Inner peace) and E3 (Optimism)). In other words, anxiety symptoms not only are influenced by other symptoms within our comorbidity network but also exert a strong cascading effect on emotional stability. This finding aligns with previous research, indicating that the influence of anxiety symptoms on emotional stability exhibits significant directionality, whereby an exacerbation of anxiety symptoms directly leads to a decline in emotional stability, thereby establishing a vicious cycle (de Voogd et al., 2017). According to Gross’s process model of emotion regulation, this bridge pathway most likely represents a failure in early-stage anxiety regulation, resulting in a negatively biased cognitive appraisal system that may eventually crystallize into a dispositional lack of emotional stability (Gross, 2002). This discovery provides a novel perspective for understanding the dynamic relationship between anxiety symptoms and emotional stability and further underscores the intervention value of mindfulness for improving both anxiety and emotional stability.

Our findings provide substantial empirical support for the emotion regulation process model by clarifying the temporal relationships among emotional stability, mindfulness, and anxiety symptoms. The findings indicate that psychological interventions ought to augment mindfulness training, particularly in the dimensions of “Thought acceptance” and “Full activity absorption,” to strengthen emotional stability and mitigate the potential exacerbation of anxiety over time. This study utilizes the CLPN approach to overcome the limits of conventional cross-sectional research and to tackle the gap in modeling the dynamic interactions among anxiety, emotional stability, and mindfulness at the item level.

## 5 Limitation and future research

This research, while providing useful insights into the temporal relationships among anxiety, emotional stability, and mindfulness, has several limitations. First, this study relied on a convenience sample drawn from a single public university in Sichuan Province, China. The sample was composed exclusively of college students within a narrow age range (*M* = 20.11, *SD* = 1.20), and therefore the observed network dynamics may not generalize to non-student populations, older adults, clinical populations with diagnosed anxiety disorders, or individuals from different cultural or socioeconomic backgrounds. The network structure may also be influenced by institution-specific contextual factors such as academic calendar, campus mental health resources, and regional cultural norms. Future research should replicate these analyses using multi-site, socio-demographically diverse, and clinical or community-based samples.

Second, data were collected at one-month intervals (May, June, July 2025) to balance capturing rapid fluctuations in anxiety and state mindfulness with allowing detectable change in the more stable trait of emotional stability (Snippe et al., 2015). This interval may not optimally capture all three constructs’ dynamics simultaneously. Future research should employ both shorter intervals (e.g., experience sampling) and longer follow-up periods.

Third, the use of brief instruments (GAD-2 for anxiety and a 3-item Emotional Stability Scale) constrained the number of network nodes for these constructs, which may limit the representativeness of content coverage and the interpretability of within-construct network dynamics. Future studies should consider employing more comprehensive measures (e.g., GAD-7) to enable richer item-level network modeling. Fourth, mindfulness and emotional stability may share variance through their common links to emotion regulation. Although the present study demonstrated their differential temporal roles within the network—with mindfulness items functioning as temporal antecedents and emotional stability items as temporal consequences—future research employing discriminant validity analyses or bifactor models may further clarify the degree of construct overlap.

Moreover, subsequent research could integrate additional variables such as individual coping styles and social support (Zimmer-Gembeck et al., 2021) to provide a more comprehensive understanding of the network dynamics. Future research could also enhance understanding by integrating the CLPN methodology with alternative approaches to clarify more complex network dynamics within the mental health ecosystem.

## 6 Conclusion

This research presents the initial evidence for the item-level temporal dynamics connecting anxiety, emotional stability, and mindfulness through a Cross-Lagged Panel Network (CLPN) approach. The findings suggest that the mindfulness dimensions of “Thought acceptance” and “Full activity absorption” are essential for improving emotional stability and reducing anxiety, while anxiety symptoms significantly affect emotional stability, thereby reinforcing the theoretical propositions of the process model of emotion regulation. These findings inspire novel intervention options for anxiety, highlighting the effectiveness of mindfulness-based training in potentially mitigating the risk of anxiety.

## Data Availability

The raw data supporting the conclusions of this article will be made available by the authors, without undue reservation.

## Footnotes

1
https://www.wjx.cn
