# Supplementary material for: Dynamic interactions among anxiety, emotional stability, and mindfulness: a cross-lagged panel network analysis
Source: Front Psychol. 2026 Jul 21;17:1740018. doi: 10.3389/fpsyg.2026.1740018 (PMC13433435; doi:10.3389/fpsyg.2026.1740018)
Supplement: Supplementary file 1 [file Supplementary_file_1.docx]

**Supplementary Material**

# **Supplementary Tables**

**Supplementary Table 1. Centrality estimates for the T1 -> T2 and T2 -> T3 cross-lagged panel networks (*z* values).**

| **Node** | **T1→ T2** | **T1→ T2** | **T2→ T3** | **T2→ T3** |
| --- | --- | --- | --- | --- |
|  | out-EI | in-EI | out-EI | in-EI |
| E1 | -0.34 | -0.45 | -0.41 | -1.27 |
| E2 | -0.33 | -0.54 | -0.98 | -0.72 |
| E3 | -0.09 | -0.85 | -0.06 | -1.28 |
| M1 | 1.12 | 0.36 | -0.09 | 0.67 |
| M2 | 1.20 | 0.02 | 0.43 | 0.38 |
| M3 | -0.47 | 1.40 | -0.02 | 1.23 |
| M4 | -0.32 | 1.23 | 0.04 | 1.14 |
| M5 | 0.93 | -0.11 | -0.52 | 0.41 |
| M6 | -0.73 | 1.31 | 2.45 | 0.73 |
| M7 | 2.18 | -0.07 | -0.27 | 0.92 |
| M8 | -0.57 | 0.61 | -0.35 | 0.25 |
| M9 | -0.58 | 0.32 | 1.79 | 0.32 |
| A1 | -1.68 | -1.01 | -1.01 | -1.76 |
| A2 | -0.32 | -2.23 | -1.00 | -1.01 |

**Note.** E1 = Not concerned with trivialities, E2 = Inner peace, E3 = Optimism, M1 = Conscious task engagement, M2 = Full task focus, M3 = Present-moment focus, M4 = Present-moment attention, M5 = Openness to experience, M6 = Full activity absorption, M7 = Thought acceptance, M8 = Nonjudgmental awareness, M9 = Acceptance of performance, A1 = Nervousness, A2 = Uncontrollable worrying.

**Supplementary Table 2. Adjacency matrix values for the T1 -> T2 cross-lagged panel network (*N* = 547).**

|  | E1 | E2 | E3 | M1 | M2 | M3 | M4 | M5 | M6 | M7 | M8 | M9 | A1 | A2 |
| --- | --- | --- | --- | --- | --- | --- | --- | --- | --- | --- | --- | --- | --- | --- |
| E1 | 1.27 | 1.12 | 1.06 | 1.00 | 1.00 | 1.00 | 1.00 | 1.00 | 1.00 | 1.00 | 1.00 | 1.00 | 0.90 | 1.00 |
| E2 | 1.16 | 1.26 | 1.17 | 0.99 | 1.00 | 1.00 | 1.00 | 1.00 | 1.00 | 1.00 | 0.99 | 0.99 | 0.92 | 0.88 |
| E3 | 1.13 | 1.16 | 1.30 | 1.00 | 1.00 | 1.00 | 1.00 | 1.00 | 1.02 | 1.00 | 1.00 | 1.00 | 0.97 | 0.90 |
| M1 | 1.00 | 1.00 | 1.00 | 1.20 | 1.12 | 1.12 | 1.08 | 1.07 | 1.11 | 1.05 | 1.02 | 1.07 | 1.00 | 1.00 |
| M2 | 1.00 | 1.00 | 1.00 | 1.05 | 1.29 | 1.19 | 1.24 | 1.00 | 1.18 | 1.00 | 1.00 | 1.00 | 1.00 | 1.00 |
| M3 | 1.00 | 1.00 | 1.00 | 1.00 | 1.00 | 1.00 | 1.02 | 1.00 | 1.00 | 1.00 | 1.00 | 1.00 | 1.02 | 1.00 |
| M4 | 1.00 | 1.00 | 1.00 | 1.00 | 1.00 | 1.02 | 1.05 | 1.00 | 1.00 | 1.00 | 1.06 | 1.02 | 1.01 | 1.00 |
| M5 | 0.98 | 1.00 | 1.00 | 1.13 | 1.06 | 1.07 | 1.00 | 1.35 | 1.05 | 1.15 | 1.00 | 1.12 | 1.00 | 1.00 |
| M6 | 1.00 | 1.00 | 1.00 | 1.00 | 1.00 | 1.00 | 1.02 | 1.00 | 1.04 | 1.00 | 1.00 | 0.93 | 1.00 | 1.00 |
| M7 | 1.00 | 1.00 | 1.00 | 1.11 | 1.04 | 1.08 | 1.09 | 1.13 | 1.10 | 1.29 | 1.26 | 1.22 | 1.00 | 1.00 |
| M8 | 0.99 | 1.00 | 1.00 | 1.00 | 1.00 | 1.00 | 1.00 | 1.00 | 1.00 | 1.01 | 1.14 | 1.00 | 1.00 | 1.00 |
| M9 | 1.00 | 1.00 | 1.00 | 1.00 | 1.00 | 1.00 | 1.00 | 1.00 | 1.00 | 1.00 | 1.00 | 1.17 | 1.00 | 1.00 |
| A1 | 0.86 | 0.85 | 0.87 | 1.00 | 1.00 | 1.00 | 1.00 | 1.00 | 1.00 | 1.00 | 1.00 | 1.00 | 1.18 | 1.01 |
| A2 | 1.00 | 0.98 | 0.97 | 1.00 | 1.00 | 1.00 | 1.00 | 1.00 | 1.00 | 1.00 | 1.00 | 0.94 | 1.21 | 1.32 |

**Note.** E1 = Not concerned with trivialities, E2 = Inner peace, E3 = Optimism, M1 = Conscious task engagement, M2 = Full task focus, M3 = Present-moment focus, M4 = Present-moment attention, M5 = Openness to experience, M6 = Full activity absorption, M7 = Thought acceptance, M8 = Nonjudgmental awareness, M9 = Acceptance of performance, A1 = Nervousness, A2 = Uncontrollable worrying.

**Supplementary Table 3. Adjacency matrix values for the T2 -> T3 cross-lagged panel network (*N* = 547).**

|  | E1 | E2 | E3 | M1 | M2 | M3 | M4 | M5 | M6 | M7 | M8 | M9 |
| --- | --- | --- | --- | --- | --- | --- | --- | --- | --- | --- | --- | --- |
| E1 | 1.34 | 1.12 | 1.11 | 1.00 | 1.00 | 1.00 | 1.00 | 1.00 | 1.00 | 1.00 | 1.00 | 1.00 |
| E2 | 1.00 | 1.13 | 1.03 | 1.00 | 1.00 | 1.00 | 1.00 | 1.00 | 1.00 | 1.00 | 1.00 | 1.03 |
| E3 | 1.15 | 1.16 | 1.28 | 1.00 | 1.00 | 1.00 | 1.00 | 1.00 | 1.00 | 0.99 | 1.00 | 1.00 |
| M1 | 1.00 | 0.95 | 0.95 | 1.09 | 1.00 | 1.04 | 1.05 | 1.00 | 1.07 | 1.01 | 1.04 | 1.09 |
| M2 | 1.01 | 1.05 | 1.00 | 1.08 | 1.22 | 1.06 | 1.13 | 1.00 | 1.15 | 1.00 | 1.00 | 1.00 |
| M3 | 1.00 | 1.08 | 1.05 | 1.05 | 1.00 | 1.00 | 1.07 | 1.00 | 1.00 | 1.00 | 1.00 | 1.01 |
| M4 | 1.11 | 1.15 | 1.12 | 1.00 | 1.00 | 1.03 | 1.00 | 1.00 | 1.00 | 1.00 | 1.00 | 1.00 |
| M5 | 0.94 | 0.83 | 0.87 | 1.11 | 1.00 | 1.00 | 1.04 | 1.25 | 1.08 | 1.13 | 1.02 | 1.00 |
| M6 | 1.01 | 1.08 | 1.10 | 1.20 | 1.26 | 1.24 | 1.18 | 1.22 | 1.09 | 1.03 | 1.00 | 1.11 |
| M7 | 1.00 | 0.92 | 0.99 | 1.00 | 1.00 | 1.00 | 1.04 | 1.06 | 1.00 | 1.08 | 1.10 | 1.04 |
| M8 | 0.99 | 0.94 | 0.88 | 1.00 | 1.00 | 1.00 | 1.00 | 1.07 | 1.01 | 1.14 | 1.22 | 1.08 |
| M9 | 1.00 | 1.14 | 1.16 | 1.00 | 1.09 | 1.20 | 1.05 | 1.00 | 1.14 | 1.19 | 1.15 | 1.28 |
| A1 | 0.83 | 0.82 | 0.81 | 1.00 | 1.00 | 1.00 | 1.00 | 1.02 | 1.00 | 1.00 | 1.00 | 1.00 |
| A2 | 0.89 | 0.86 | 0.87 | 1.00 | 1.01 | 1.00 | 1.00 | 1.00 | 1.00 | 1.00 | 1.00 | 1.00 |

**Note.** E1 = Not concerned with trivialities, E2 = Inner peace, E3 = Optimism, M1 = Conscious task engagement, M2 = Full task focus, M3 = Present-moment focus, M4 = Present-moment attention, M5 = Openness to experience, M6 = Full activity absorption, M7 = Thought acceptance, M8 = Nonjudgmental awareness, M9 = Acceptance of performance, A1 = Nervousness, A2 = Uncontrollable worrying.

**Supplementary Figures**

**Supplementary Figure 1. Complete cross-lagged panel networks of anxiety, emotional stability, and mindfulness, including autoregressive edges.**

**Note.** Blue edges represent positive cross-lagged associations, and red edges represent negative cross-lagged associations. The thickness of the edges represents the relative strength of the associations, with thicker edges indicating stronger relationships. E1 = Not concerned with trivialities, E2 = Inner peace, E3 = Optimism, M1 = Conscious task engagement, M2 = Full task focus, M3 = Present-moment focus, M4 = Present-moment attention, M5 = Openness to experience, M6 = Full activity absorption, M7 = Thought acceptance, M8 = Nonjudgmental awareness, M9 = Acceptance of performance, A1 = Nervousness, A2 = Uncontrollable worrying.

**Supplementary Figure 2. Accuracy of edge-weight estimation in the cross-lagged panel networks.**

**Note.** Each horizontal line represents an edge in the network. The red and gray dots represent the sample value and the bootstrap mean, respectively. The gray band represents the bootstrapped confidence interval. E1 = Not concerned with trivialities, E2 = Inner peace, E3 = Optimism, M1 = Conscious task engagement, M2 = Full task focus, M3 = Present-moment focus, M4 = Present-moment attention, M5 = Openness to experience, M6 = Full activity absorption, M7 = Thought acceptance, M8 = Nonjudgmental awareness, M9 = Acceptance of performance, A1 = Nervousness, A2 = Uncontrollable worrying.

**Supplementary Figure 3. Bootstrapped difference test for edges in the cross-lagged panel networks.**

**Note.** Gray boxes indicate no significant difference between edge weights, whereas black boxes indicate significant differences. E1 = Not concerned with trivialities, E2 = Inner peace, E3 = Optimism, M1 = Conscious task engagement, M2 = Full task focus, M3 = Present-moment focus, M4 = Present-moment attention, M5 = Openness to experience, M6 = Full activity absorption, M7 = Thought acceptance, M8 = Nonjudgmental awareness, M9 = Acceptance of performance, A1 = Nervousness, A2 = Uncontrollable worrying.

**Supplementary Figure 4. Stability of centrality metrics in the cross-lagged panel networks.**

**Note.** The x-axis represents the percentage of sampled cases, and the y-axis represents the average correlation with the original sample. The line represents the mean, and the shaded area represents the range from the 2.5th to the 97.5th percentile.

**Supplementary Figure 5. Bootstrapped difference test for in-expected influence of nodes in the cross-lagged panel networks.**

Note. Gray boxes indicate no significant difference, whereas black boxes indicate significant differences (*p* < 0.05). E1 = Not concerned with trivialities, E2 = Inner peace, E3 = Optimism, M1 = Conscious task engagement, M2 = Full task focus, M3 = Present-moment focus, M4 = Present-moment attention, M5 = Openness to experience, M6 = Full activity absorption, M7 = Thought acceptance, M8 = Nonjudgmental awareness, M9 = Acceptance of performance, A1 = Nervousness, A2 = Uncontrollable worrying.

**Supplementary Figure 6. Bootstrapped difference test for out-expected influence of nodes in the cross-lagged panel networks.**

**Note.** Gray boxes indicate no significant difference, whereas black boxes indicate significant differences (*p* < 0.05). E1 = Not concerned with trivialities, E2 = Inner peace, E3 = Optimism, M1 = Conscious task engagement, M2 = Full task focus, M3 = Present-moment focus, M4 = Present-moment attention, M5 = Openness to experience, M6 = Full activity absorption, M7 = Thought acceptance, M8 = Nonjudgmental awareness, M9 = Acceptance of performance, A1 = Nervousness, A2 = Uncontrollable worrying.
